# Trans-Radial Approach: technical and clinical outcomes in neurovascular procedures

**DOI:** 10.1186/s42155-020-00152-z

**Published:** 2020-10-08

**Authors:** D. G. Romano, G. Frauenfelder, S. Tartaglione, F. Diana, R. Saponiero

**Affiliations:** Department of Neuroradiology, A.O.U. San Giovanni di Dio e Ruggi d’Aragona, Via San Leonardo 1, 84100 Salerno, Italy

**Keywords:** Transradial approach, Neuroradiography, Digital subtraction angiography, Endovascular procedure

## Abstract

**Background:**

To evaluate efficacy and safety of Trans-Radial Approach (TRA) in cerebral angiography for diagnostic and therapeutic purpose.

**Methods:**

We retrospectively included consecutive patients eligible for TRA cerebral angiography at our Institution between September 2019 and January 2020. Cerebral DSA was classified in *diagnostic* (one-vessel imaging) or *therapeutic* (emergency/elective). Technical and clinical outcome were recorded for each group.

**Results:**

A total of 61 TRA angiographies were evaluated. Right-sided TRA was obtained in 85,2% of all cases. Interventional procedures included 11 strokes, 2 ruptured aneurysms, 2 unrupted aneurysms, 1 DAVF and 3 symptomatic atheromatous intracranial stenosis. Successful TRA angiographies were obtained in 97,6% and 94,7% for diagnostic and therapeutic group, respectively. No major radial artery complications were recorded. Mean puncture-to-final angiogram was 11 and 62 min for diagnostic and therapeutic groups, respectively. Mean radial compression maintenance was 4 h, allowing patients discharge within 6 h in all cases undergone diagnostic angiography.

**Conclusions:**

TRA could be a valid technique in terms of efficacy and safety both for diagnostic and therapeutic cerebral angiographies, with low complication rate.

## Introduction

In the cardiology literature, benefits of Trans-Radial Approach (TRA) compared with traditional Trans-Femoral Approach (TFA) have been well reported (Bertrand et al. 2012). Additionally, TRA for cerebral angiography has been described in several retrospective series and shown to be safe and effective (Mitchell et al. 2012; Jo et al. 2010), due to a reduction in access site complications, decreased length of stay, reduced hospital costs (Snelling et al. 2018a), and improved patient satisfaction (Mann et al. 1996).

Nonetheless, use of TRA as the primary method of performing cerebral angiography has not been widely adopted. There are several obstacles to transitioning a cerebral angiography practice to TRA, including attitude with access and vessel selection, longer learning curve for the operator (Liu et al. 2019) and concerns over safety and technical feasibility of transition to practice.

Aim of our study was to evaluate efficacy and safety of TRA for cerebral angiography in cerebrovascular procedures.

## Materials and methods

### Patients selection

From September 2019 to January 2020, we retrospectively evaluated all consecutive patients who were eligible for TRA and underwent diagnostic or therapeutic DSA at our Institution. Inclusion criteria were:

- age ≥ 18 years;
- palpable radial artery pulse;
- diagnostic angiography requiring one-vessel imaging;
- therapeutic endovascular procedure with vertebro-basilar etiology or marked supra-aortic branch tortuosity (evaluated at CT-Angiography or during DSA);
- TFA failure in emergency setting.

Informed consent to participate in the study was obtained from all patients.

### Cerebral angiography technique

The right arm approach was adopted in every case except for the intentional left TRA used for left vertebra-basilar imaging or after right TRA failure in cases requiring one-vessel cerebral angiography.

Patient demographics, clinical data and angiographic metrics were recorded.

Diagnostic and therapeutic DSA were performed using monoplane suite (AlluraClarity - Xper FD 20, Philips) in a supine patient position with the arm adducted to the hip. Historically, Allen’s or modified Allen’s test has been described for assessing collateral circulation of the hand, but has recently been demonstrated to be unnecessary so our protocol did not involve performing this test (Valgimigli et al. 2014; Bertrand et al. 2014).

The radial artery area was sterilized and draped. The peri-arterial tissue was infiltrated with 1 mL 1% lidocaine, and the radial artery was usually cannulated using a 21 G needle with counter puncture technique then using a 11 cm 4- or 5-F radial kit introducer (AVANTI® + Introducer, Cordis, Santa Clara, CA, USA). In our cases, no ultrasound guidance was used. Antispasmodic agent (verapamil 5 mg/10 mL) was administered intra-arterially through the side-port of the introducer, to prevent radial artery spasm. At the end of cerebral DSA, hemostasis was achieved using a radial compression device (*TR BAND®* - Terumo, Somerset, New Jersey, USA). The band was gradually deflated after 1 or 2 h depending on patient anticoagulant/antiplatelet therapy. If bleeding arrest was confirmed, bend was removed applying sterile dressing. Before discharge, radial artery occlusion or other local complications were checked by local palpation and by reverse Barbeau test.

Diagnostic angiographies with right radial access were usually performed with a 5-F Simmon I catheter (Cordis, Santa Clara, CA, USA) for ipsilateral CCA studies and with a 5-F Vertebral catheter (Cordis, Santa Clara, CA, USA) exchanged on a 260 cm-wire (Radifocus, Terumo medical, Somerset, New Jersey, USA) for selective ipsilateral ICA angiography or directly with a 5-F Simmon II catheter to value CCA or ICA. In left radial access, CCA/ICA catheterization was usually performed with 5-F Simmon II catheter. For VA catheterization, a 5-F Vertebral catheter was usually used. Catheter selection was also based on operator experience and aortic arch features (also evaluated on previous CT-angiography).

For therapeutic DSA, TRA was exploited both for single access and in adjunction to TFA, used for infra-procedural angiographic checks. For therapeutic DSA, exchange with a 6-F introducer or a 7,5-F Sheatless catheter was obtained. A coaxial system was used most of the time. Occasionally, tri-axial system was required, including a distal/reperfusion catheter and a microcatheter.

Three-dimensional (3D) reconstruction of rotational angiography was obtained when needed.

### Cerebral angiography classification and recorded data

Trans-radial approach for cerebral DSA was retrospectively classified in two groups:

- *diagnostic*: requiring one-vessel imaging (the intended large vessel of interest), as first study or treatment follow-up;
- *therapeutic:* including emergency and elective procedures.

Radial access side, introducer size, diagnostic and/or procedural catheters used, successful TRA access (reported as vessel-of-interest catheterization), number of cannulated supra-aortic arteries, radial-related peri-procedural complications (radial vasospasm or occlusion, local hematoma, artery rupture), procedural time (from puncture to final angiogram) were recorded. Radial compression maintenance (from bend application to removal) was recorded only for diagnostic group (as patients were on outpatients basis), while technical procedure success rate was recorded in the therapeutic one.

### Statistical analysis

Data are presented as mean for continuous variables and as frequency (percentages) for categorical variables.

## Results

A total of 64 cerebral angiography in 61 patients (36 women), with a mean age of 63,2 years (range 31–87) were retrospectively evaluated. Three patients underwent cerebral DSA twice in the study period. In 3 cases access over the radial artery was not achieved (radial loop/tortuosity) and TFA was required because of left internal carotid artery (ICA) to be catheterized: one case of AIS with left M1 segment occlusion, one case of left atypical hematoma and one case of diagnostic follow-up for treated left middle cerebral artery (MCA) aneurysm.

**Fig. 1:**
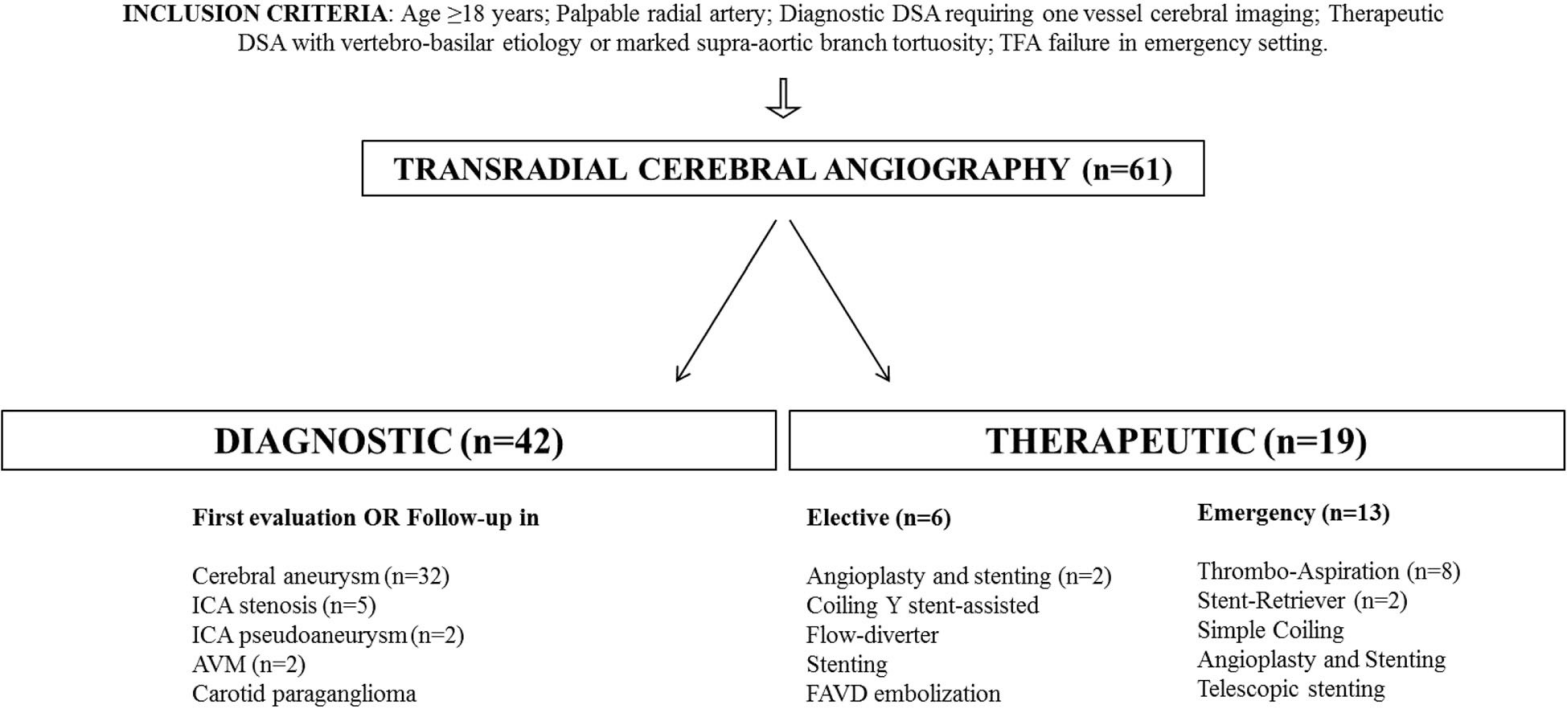
Schematic representation for inclusion criteria and cerebral angiography classification in patients undergoing trans-radial access

A total of 61 TRA angiography were obtained and included in the final study (Fig. 1). Right-sided TRA was obtained in 85,2% (*n* = 52/61) cases, while left-sided in 14,8% (*n* = 9/61): in the latter, two cases for intentional access due to AIS of left vertebro-basilar segment; in the other 7 cases for *diagnostic* DSA after right radial failure.

### Diagnostic group

TRA DSA requiring one-vessel catheterization was obtained in 68,9% of cases (*n* = 42/61): 32/42 cases for imaging follow-up; 10/42 cases for first angiographic diagnosis. Imaging was obtained for cerebral aneurysm diagnosis or follow-up (*n* = 32), ICA stenosis (*n* = 5), ICA pseudoaneurysm (*n* = 2), treated AVM (*n* = 2) and carotid paraganglioma (*n* = 1).

Vessel of interest was catheterized in 41/42 (97,6%) cases (*n* = 4 right CCA; *n* = 1 left CCA; *n* = 22 right ICA; *n* = 7 left ICA; *n* = 5 right VA; *n* = 2 left VA); in one case, the right ICA could not be catheterized due to significant vessel tortuosity and the exam crossed over TFA.

**Fig. 2:**
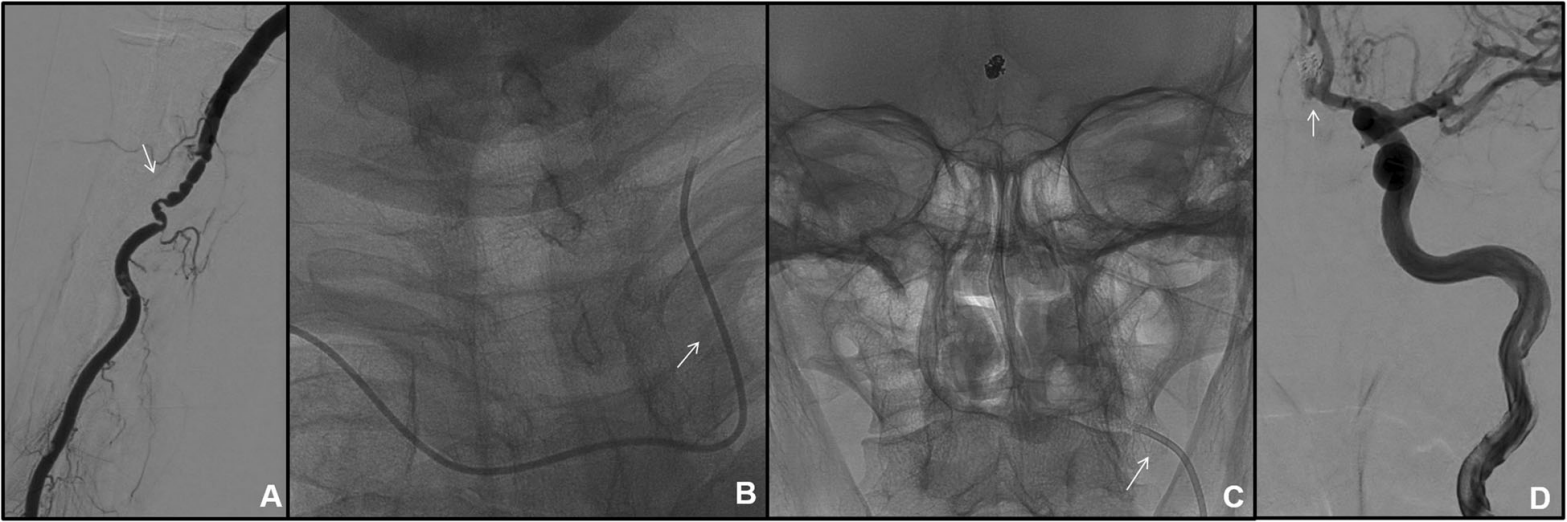
Follow-up cerebral DSA for ACoA aneurysm treated with simple coiling. Small right radial lumen and vasospasm (arrow in **a**) required a 4-F introducer kit; a 4-F SIM 1 catheter was advanced through the aortic arch into the left ICA (arrow in **b** and **c**); selective left ICA angiography demonstrated no residual aneurysm (arrow in **d**). Left ICA catheterization was necessary because of right A1 segment hypoplasia.

**Fig. 3:**
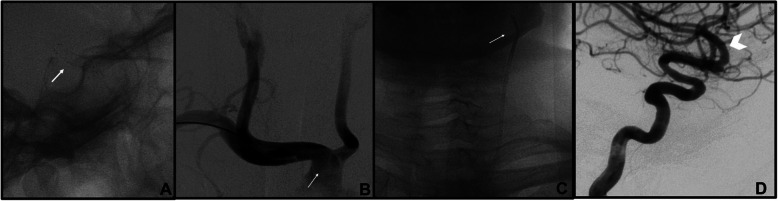
Follow-up cerebral DSA for left para-ophtalmic ICA aneurysm treated with a FD stent (arrow in **a**) in a 47-year-old woman. Presence of bovine aortic arch (**b**) permitted right TRA and selective left ICA catheterization (**c**) with a 5 Fr Vertebral catheter to evaluate treatment efficacy (**d**)

In a total of 34/41 (82,9%) right-sided TRA angiographies, the ipsilateral CCA catheterization (*n* = 4/34) was performed with Simmon I in 3 cases and with Simmon II 2 in one case; the ipsilateral ICA (*n* = 22/34) was selected with 5Fr Vertebral catheter in 3 cases, on exchange wire in 19 cases. Contralateral left CCA/ICA catetherization (*n* = 7) was obtained with 5 Fr Simmon II catheter in 5 cases and with a 4-F Simmon I catheter because of radial artery spasm in one case (Fig. 2). One left CCA was performed with 5-F Vertebral catheter because of bovine aortic arch (Fig. 3). For VA catheterization (*n* = 5), a 5-F Vertebral catheter was always used, excepted in one case in which a 4-F was chosen because of small radial artery size. In a total of 7/41 (17,1%) left-sided TRA angiographies was obtained after right failure (2 cases for previous occlusion and 5 cases for difficult to cannulate). In all cases, contralateral ICA catheterization was performed with 5-F Vertebral catheter.

A median of 3 (range 1–6) supra-aortic arteries were cannulated for each diagnostic cerebral angiography. All diagnostic TRA angiographies were performed in an outpatients basis: mean radial compression maintenance was 4 h (range 3–5,5). Patients were dismissed within 6 h after radial puncture in all cases.

### Therapeutic group

**Fig. 4:**
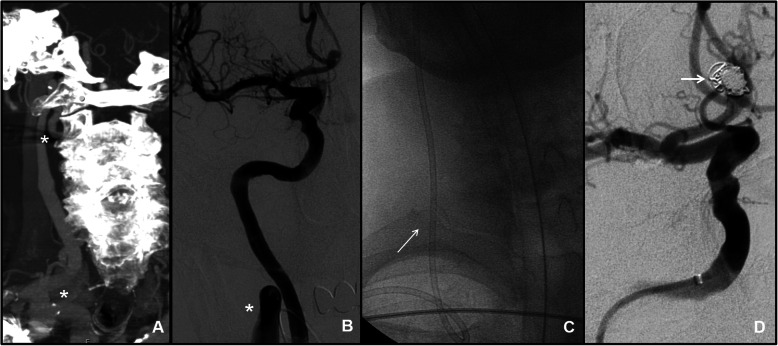
Sub-arachnoid hemorrhage with ACoA aneurysm in a 76-year-old man. Right trans-radial access was chosen because of a double kinking at CCA and ICA origin evaluated at CT-angiography and during DSA (asterisks in **a** and **b**). A 6 Fr guiding catheter was advanced for distal access into proximal ICA and simple coiling was obtained with compete aneurysmal occlusion (arrow in **d**)

**Fig. 5:**
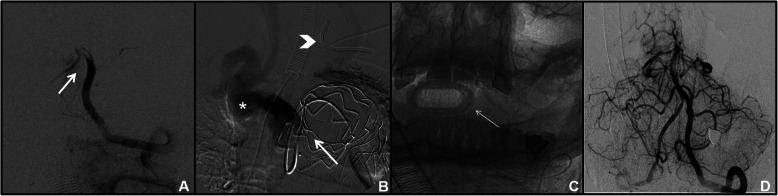
Left TRA in a 64-year-old woman with AIS of left vertebro-basilar segment (arrow in **a**). After left VA catheterization with a 6-F guiding catheter (arrowhead in **b**), additional femoral approach for right procedural checks was not achieved (arrow in **b**) because of marked brachiocephalic tortuosity (asterisk in **b**) and aortic endoprosthesis. After a first-pass mechanical thrombectomy with stent retriever (arrow in **c**) a TICI 3 was obtained (**d**)

*Therapeutic* DSA was obtained in 31,1% (*n* = 19/61) of all cases. Emergency procedures (*n* = 13) included 11 cases of AIS and 2 cases of SAH in ruptured aneurysm (Fig. 4). Elective procedure (*n* = 6) included two aneurysms, one FAVD and three intracranial atheromatous stenosis treatment. Type of lesion and technical data are described in Table 1**.** Right-sided radial access was obtained in 17/19 cases, while intentional left TRA due to posterior circulation AIS was obtained in 2/19 cases (Fig. 5). Vessel-of-interest was catheterized in 94,7% (*n* = 18/19) of cases. In one case, procedure was not completed as catheterization of VA was impeded from both femoral and radial access by marked angulated vessel origin.

**Table 1:**
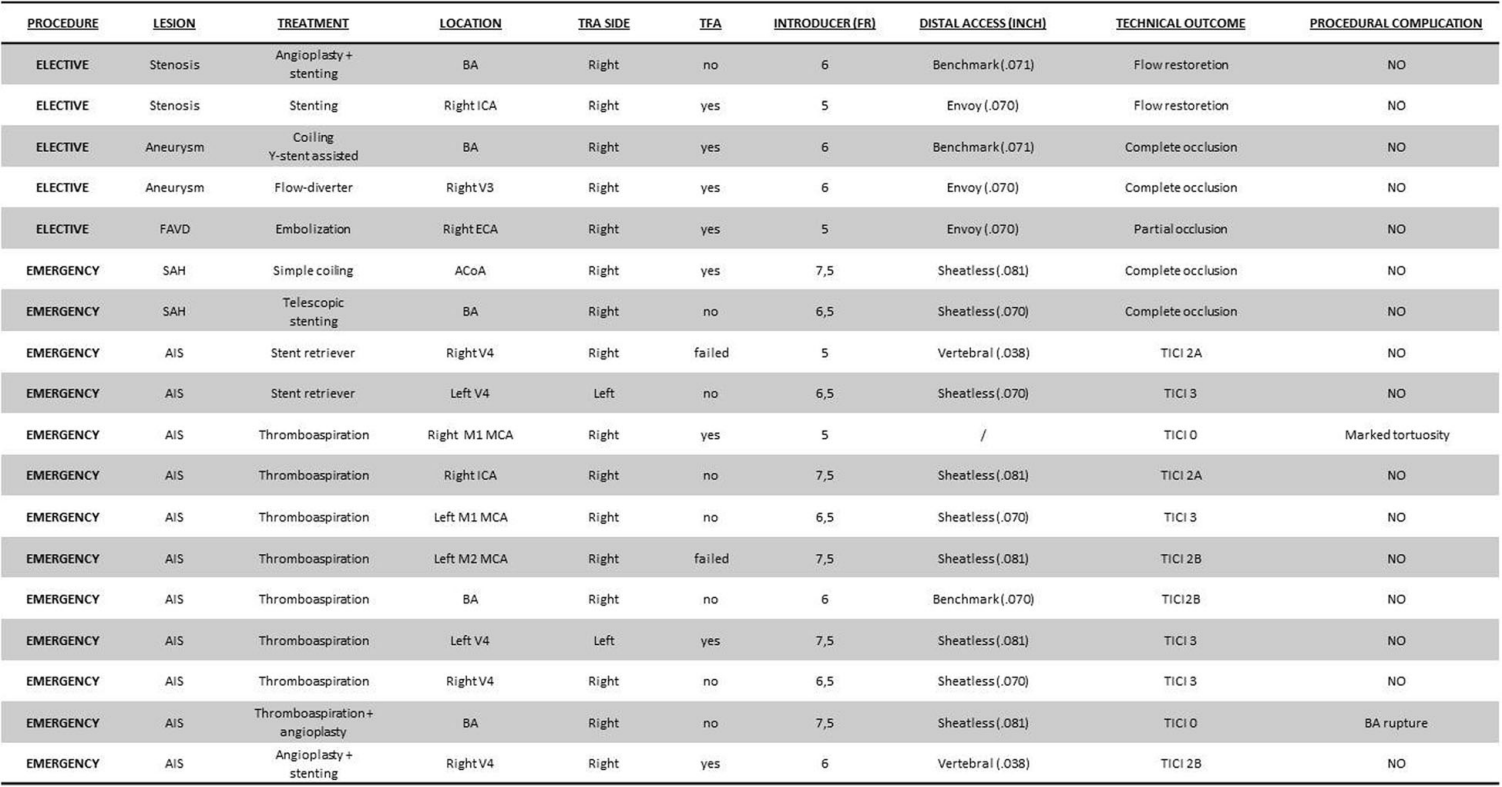
Clinical, technical features and complications in the therapeutic group. (Legend: TRA: Trans-radial access; FAVD: Dural Arteriovenous fistula; BA: Basilar Artery; MCA: Middle Cerebral Artery; ACoA: Anterior Communicating Artery; VA: Vertebral Artery; TICI: Thrombolysis in cerebral infarction scale)

**Fig. 6:**
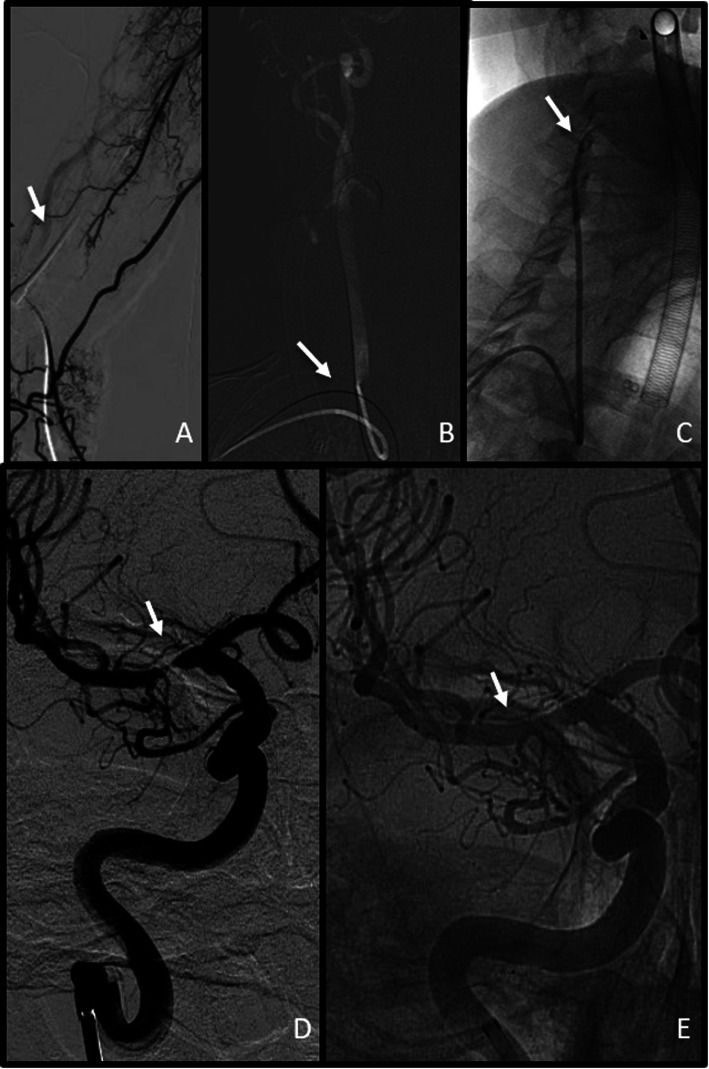
Therapeutic DSA in a in a 72-year-old man with right intracranial atheromatous stenosis. After 6-F right TRA (arrow in **a**), on exchange wire in right ECA (arrow in **b**), a 95-cm Benchmark .071 was advanced into right ICA (arrow in **c**) and right M1 MCA flow restoration was obtained after angioplasty and stenting (arrows in **d** and **e**).

Two right TRAs were conducted after TFA failure; two elective procedures were accompanied by TFA for infra-procedural angiographic checks. A 5-F introducer was used in all cases. After selecting the target vessel, a long exchange for a 6F guiding catheter (*Envoy 070* guide catheter, Codman Neuro, Raynham, Massachusetts, USA; *Benchmark 071* Delivery catheter, Penumbra Inc., Alameda, CA, USA) or a 7,5-F sheatless catheter (*Asahi* Intecc Co Ltd., Tustin, CA, USA) was performed using a 260-cm wire (Radifocus, Terumo, Somerset, New Jersey, USA) (Fig. 6).

Procedural complications were recorded in 1/19 cases, due to basilar artery rupture after angioplasty for basilar AIS. Technical success of procedural angiographies is reported in Table 1.

In all sample, radial complications were recorded in 3/64 cases (4,7%): two cases due to artery occlusion and one case due to vasospasm. No local hematoma or artery rupture were recorded. Anatomical variant with bovine aortic arch was present in two patients (3,3%). Mean puncture-to-final angiogram was 11 min (range 4–25) and 62 min (range 40–115) respectively for diagnostic and procedural groups.

## Discussion

We reported our initial single-center experience with TRA for diagnostic and therapeutic DSA in interventional neuroradiology on 61 consecutive angiographies. To our knowledge, only one study describing TRA experience as the frontline approach for both diagnostic and interventional procedures over a short time course has been published (Almallouhi et al. 2020). We have introduced this alternative approach for cerebral angiography for less than a year and our results demonstrated that TRA cerebral angiography could be a valid alternative to TFA; no major complications occurred and a low rate of minor complications (4,7%) concerning the access site were registered. Two cases of asymptomatic radial occlusion were recorded at the beginning of our training experience, maybe due to bend error in decompression timing. In previous studies, compression time and pressure have been shown to contribute to radial artery occlusion (Cubero et al. 2009); moreover, radial complication events (puncture failure, artery spasm and occlusion) would be less frequent as the interventionist gains experience (Liu et al. 2019; Brunet et al. 2020). In fact, Snelling et al. *(*Snelling et al. 2018b*)* demonstrated that the learning curve of the radial access rapidly improve between case 5 and case 15 in a neurointerventional scenario.

Successful TRA angiographies as vessel-of-interest catheterization was obtained in 97,6% and 94,7% for diagnostic and therapeutic group, respectively, as various recent studies documented (92.2–98.6%) (Jo et al. 2010; Snelling et al. 2018b; Matsumoto et al. 2001; Park et al. 2013).

Right radial puncture was preferred over the left side (85,2% vs 14,8%): this was adopted for bilateral CCA and ICA selection, using Vertebral and/or Simmons (I, II) catheters. An exchange wire is recommended when the type of catheter needs to be changed to avoid recrossing of the vessels and to mount the co-axial system. Although left TRA is rarely reported for cerebral angiography, interventional cardiologists have reported extensive experience. The most commonly perceived pitfalls for left transradial access include increased procedural time and operator discomfort (Barros et al. 2020). In our sample, left-sided radial access was preferred in posterior circulation or in case of right puncture failure; crossing over the left radial artery may contributed to reduce the number of cases converted in TFA (3,2%) in comparison to other published studies (4,7%- 24,8%) (Almallouhi et al. 2020; Snelling et al. 2018b; Joshi et al. 2020). An important aspect of our study was that patients undergone diagnostic DSA were in the outpatient setting, providing advantage in dismission time (allowing the patient to ambulatory immediately after the procedure); it is well-established that observation time after TRA for cerebral angiography is shortened (max. 4 h vs. 6–24 h after TFA) resulting in a reduction of nursing workload and hospital costs. Prospectively, cerebral angiographies before or after neurointerventional procedures may be easily performed on ambulatory basis. Three-dimensional (3D) reconstruction of rotational angiography significantly improved the performance of DSA in diagnosis of small aneurysms (which could not be seen on CTA and MRA) and for an accurate pre-treatment planning. The 3D DSA was proposed to become a new gold standard of interventional cerebral vascular imaging due to its high spatial resolution with 3D imaging and dynamic information. In therapeutic procedures, we evaluated TRA in case of marked supra-aortic vessel tortuosity, TFA failure and/or posterior circulation lesions and also in obese patients. Interventional procedures normally require a relatively high catheter/sheath placement and therefore TRA has been considered not ideal because of the relatively small size of the radial artery, smaller than a 7-F introducer sheath in almost one-third of the men and in two-thirds of the women (Saito et al. 1999). The sheathless TRA technique could solve this problem and prevent access site complications: we used a 7,5 Fr Sheathless Eaucath system (*Asahi* Intecc Co Ltd.) in four AIS treated with thromboaspiration and in one case of ruptured aneurysm treatment to allow large-bore distal access. This system provides an integrated introducer and proprietary guiding catheter (0.081-in. internal diameters; 6-F outer diameter), and its hydrophilic coating allows a very smooth entry into the vessel, especially if preceded by a small 5-F sheath (Cheaito et al. 2015). Heretofore, sheatless system have been reported in only case of mechanical thrombectomy in neurointerventional field (McCarthy et al. 2019). The 6-F Benchmark has been another guide catheter of choice for our interventional cases as it fits in a 6-F sheath as opposed to other guide catheters we usually use in TFA (eg, Neuron Max 088; Penumbra, California, USA) or AXS Infinity (Stryker, California, USA) which require an 8-F sheath.

Mean puncture-to-final angiogram was 62 min in interventional procedures of our study: a recent systematic review (Joshi et al. 2020) reported no significant differences in mean access to reperfusion time in patients undergoing mechanical thrombectomy or via TFA or TRA (61.9 vs 61.1 min). TRA may be particularly well suited for stroke patients whose aortic arch anatomy would present a challenge using TFA. Therefore, in cases of planned interventions through the left vertebral artery, we performed TRA from the left side.

Our initial experience with TRA suggests that a busy neurointerventional practice can overcome the basic radial learning curve in several months and after operators each perform about 60 cases, as reported by Zussman et al. (Zussman et al. 2019).

Moreover, TRA results the preferred access in a majority of patients, especially those who had undergone prior TFA (Snelling et al. 2018b; Satti et al. 2017).

Limitations of our study were the retrospective design and the small sample size. Moreover, absence of an ultrasound-guided radial puncture, which could eliminate access failure and could improve efficiency. Larger-scale studies are needed to confirm our initial findings.

## Conclusions

Initial experience with the TRA for diagnostic and therapeutic neurointerventional angiographies demonstrated that this could be a valid technique in terms of efficacy and safety, with a low complication rate, even over a short time course. With increasing familiarity, development of TRA-specific neuroendovascular devices, TRA is expected to become more widely used by neurointerventionalists.

## Data Availability

The datasets generated and/or analysed during the current study are not publicly available due to privacy reason but are available from the corresponding author on reasonable request.
